# Development and validation of self-efficacy and intention measures for spending time in nature

**DOI:** 10.1186/s40359-022-00764-1

**Published:** 2022-03-03

**Authors:** Jay E. Maddock, Courtney Suess, Gregory N. Bratman, Carissa Smock, Debra Kellstedt, Jeanette Gustat, Cynthia K. Perry, Andrew T. Kaczynski

**Affiliations:** 1 Department of Environmental and Occupational Health, School of Public Health, Texas A&M University, 1266 TAMU, College Station, TX 77843 USA; 2 Center for Health and Nature, Houston Methodist Research Institute, Houston, TX USA; 3 Department of Recreation, Park and Tourism Sciences, Texas A&M University, College Station, TX USA; 4 School of Environmental and Forest Sciences, University of Washington, Seattle, WA USA; 5 School of Business, Northcentral University, San Diego, CA USA; 6 Texas A&M AgriLife Extension, College Station, TX USA; 7 Department of Epidemiology, Tulane University, New Orleans, LA USA; 8 School of Nursing, Oregon Health and Sciences University, Portland, OR USA; 9 Department of Health Promotion, Education, and Behavior, University of South Carolina, Columbia, SC USA

**Keywords:** Social Cognitive Theory, Health promotion, Nature, United States

## Abstract

**Purpose:**

The purpose of this study was to develop and evaluate the reliability and validity of self-efficacy and intentions measures for time spent in nature (TSN). TSN is related to improvement in psychological well-being and health, yet most American adults spend very little time in such settings. Theory-based interventions have been effective in increasing physical activity, a related behavior, and may be one mechanism to increase TSN. Self-efficacy and intentions have been shown to be strong predictors of health behaviors and are used across several theories. However, scales to measure these factors have not yet been developed and are needed to facilitate effective interventions.

**Methods:**

TSN self-efficacy and intentions scales were developed using a sequential nine-step procedure: identification of the domain and item generation; content validity; pre-testing of questions; sampling and survey administration; item reduction; extraction of factors; tests of dimensionality; tests of reliability; and tests of validity. The 14-member multidisciplinary, researcher and practitioner investigative team generated 50 unique items for self-efficacy and 24 unique items for intentions. After subjecting items to content validity and pre-testing, item sets were reduced to 21 assessing self-efficacy and nine assessing intentions. A nationwide sample of 2109 adult participants (49.7% female, Mean Age = 58.1; 59.8% White, 18.4% Hispanic, 13.3% Black) answered these items via an on-line survey.

**Results:**

Using split-half measures, principal components analysis indicated a one-factor solution for both scales. The factor structure was upheld in confirmatory factor analyses and had high internal consistency (α = .93 self-efficacy; .91 intentions). The scales were moderately correlated with each other (r = .56, *p* < .001) and were strongly related to TSN with large effect sizes (eta^2^ > .20).

**Conclusions:**

The study resulted in reliable and valid self-efficacy (14 items) and intentions (8 items) scales that can be used to develop future theory-based interventions to increase TSN and thereby improve population health.

## Introduction

### Relationship of nature to health

A body of evidence from various disciplines has demonstrated the myriad ways in which nature contact is associated with physical and mental health [1–3]. These findings include investigations of visits to a variety of different types of nature, from urban greenspace to large forests outside of city limits, residential and community gardens, ocean beaches, coastlines, and many other environments [4–7]. Documented effects and associations range from cognitive restoration to reductions in stress, anxiety, and mental health disorders, improvements in emotion regulation, enhanced immune function, increased physical activity, and social cohesion [8–13]. Many questions remain, however, about causal mechanisms, the characteristics of the dose–response relationship with respect to specific outcomes, and the ways in which individual and population-level differences may moderate the impact of nature contact on health [14–18].

### Need for interventions to increase time spent in nature

Despite the strength of this evidence and recent efforts to incorporate considerations of the health benefits of nature contact into urban planning, as well as increasing support for “green prescriptions” from some health care providers, most Americans spend less than five hours per week in nature [19]. As attention from diverse sectors turns to potential interventions that may increase nature contact, a variety of considerations must be taken into account. This includes further research into the factors that influence intentions and capability to visit natural spaces.

Connectedness to nature is a construct that is examined in a variety of different contexts within the literature, using the Connectedness to Nature Scale [20]; the Inclusion of Nature in Self Scale [21]; the Nature Relatedness Scale [22] and other measures. Increased feelings of connection to the natural world is found to be associated with well-being and pro-environmental behaviors (e.g., support for conservation) [23] and some evidence is emerging that this connection may predict frequency of nature contact as well [24]. Interventions that increase these feelings of relatedness and connectedness to the natural world may therefore have repercussions on intentions for future visitation to these environments. These types of motivations are likely to be related to intrinsic motivation for nature contact. This is an important factor to consider, as recent research has demonstrated the importance of accounting for intrinsic versus extrinsic motivation or perceived social pressure to visit nature, and how these differences may moderate the affective impacts of nature contact [25].

Despite feelings of connection to nature, however, significant obstacles exist for some individuals in the form of social, financial, and physical barriers to access [26–31] and experiences of discrimination and lack of safety within these spaces [32–35]. These barriers to access and participation are very likely to adversely impact intentions to visit nature. Park design and maintenance, amenities, neighborhood characteristics, and provision of inclusive programming are also significant predictors of use and visitation [36–39]. Access to nature is therefore determined not only by physical distance and adequate infrastructure, but by capabilities that are highly influenced by social and economic factors as well [12, 40].

### Theoretical underpinnings

Theory-based interventions are effective in changing a wide variety of health behaviors from smoking to physical activity and organ donation [41]. To date, interventions to increase time spent in nature are focused on increasing access to green space, physician-based prescriptions, and programmatic activities [42, 43]. The development of valid and reliable measures of theoretical constructs is an essential first step in developing theory-based interventions [44]. These and other studies demonstrate the need for developing valid and reliable psychosocial measures that support theory-based interventions to increase time spent in nature [2].

Self-efficacy and intentions are two of the most robust theoretical constructs in predicting behaviors. Self-efficacy was originally integrated into Social Cognitive Theory and is integrated into the Theory of Planned Behavior, Health Belief Model, and the Transtheoretical Model [45–47]. Intentions are the key construct in the Theory of Planned Behavior and the Theory of Reasoned Action. Fishbein positioned intentions as the main construct through which attitudes, norms and self-efficacy effect behaviors [48].

Self-efficacy includes perceived confidence to conduct a behavior successfully [45]. Influenced by individual capabilities and environmental factors, self-efficacy includes control over barriers as well as ability to perform a behavior [49]. Self-efficacy is shown to be one of the strongest predictors of intentions and behavior across a variety of studies [50, 51]. For example, Netz et al. found positive correlations between high levels of self-efficacy and performing physical activity, suggesting that perceived self-efficacy in ability to perform physical activity must be established before other motivational interventions are considered [52]. It is therefore probable that self-efficacy is necessary to increase spent time in nature; however, measures to determine this association are needed.

Intentions to perform a behavior are the most proximate measure to a health behavior [53]. Attitudes, norms, and self-efficacy have all been shown to influence intentions and, through changes in intentions, behavior [50, 54]. The Theory of Planned Behavior posits that behavior is influenced directly through intentions which mediate all other pathways [53]. Therefore, measuring intentions to spend time in nature is critical as changes in self-efficacy, attitudes, and norms should directly influence intentions [55].

### Study aims

The goals of this study were to develop reliable and valid scales for self-efficacy and intentions to spend time in nature.

## Methods

### Design

**Fig. 1:**
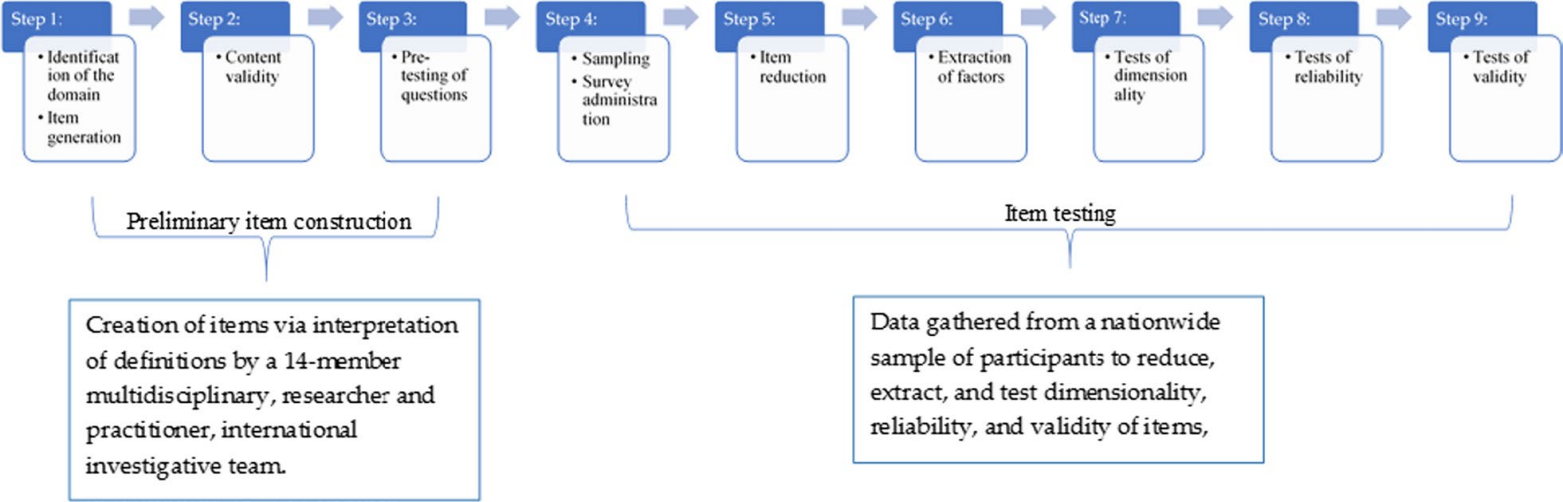
Pictorial representation of methodology steps

The TSN self -efficacy and intentions scales were developed using the sequential methods developed by Jackson [56] and Comrey [57] and expanded on by Boateng et al. [58]. These methods follow a nine-step procedure: (1) identification of the domain and item generation; (2) examination of content validity; (3) pre-testing of questions; (4) sampling and survey administration; (5) item reduction; (6) extraction of factors; (7) tests of dimensionality; (8) tests of reliability; and (9) tests of validity. Figure 1 provides a pictorial representation of these study stages.

In phase one, self-efficacy was defined as a person's confidence in his or her ability to take action and to persist in that action despite obstacles or challenges pertaining to spending time in nature. Intentions were defined as planning to engage in certain nature-related behaviors over the next three months. The 14-member multidisciplinary, researcher and practitioner, international investigative team individually generated items about self-efficacy and intentions to spend time in nature based on these prompts. For example, the intentions prompt was, “In the next three months, do you intend to…” The lead investigator reviewed all generated items and eliminated duplicates. In phase two, all items were reviewed and rated individually by the investigative team using a Qualtrics survey. Items were first rated on how relevant they were to the construct of self-efficacy or intentions on a four-point Likert scale ranging from not relevant to very relevant. Next, thematic subsets were rank ordered based on their importance to spending time in nature. Items that had means of less than 2.5 on relevance and scored in the bottom quartile of importance were removed.

In phase three, several members of the research team recruited community members across the country to participate in pretesting of items using a cognitive interview. A research team member sat with an individual as they read the questions and took item-by-item feedback for items that were confusing, double barreled, or otherwise difficult to answer [44]. Poorly preforming items were again eliminated during this phase of the study.

In phase four, a nationwide sample of participants was acquired through a panel from Qualtrics of United States (U.S.) adult residents aged 18 or older. There is some disagreement on the sample size needed for factor analysis ranging from 5 to 10 respondents per item to 100 to 1000 respondents per study. Comrey [57] has rated a sample size of 1000 as excellent for factor analysis stability. Since this study was using split-half methods, a sample of 2000 was planned. Respondents included in the Qualtrics database were self-selected to be part of the present study. To minimize self-selection bias, Qualtrics sends a survey invitation to its panel members without disclosing the particular topic so that respondents participate in the survey without knowing the nature of the survey beforehand, effectively facilitating a more random sampling procedure. Data collection was completed over a one-month period (June 23-July 21, 2021) and respondents were stratified by age, gender, and region within the U.S. to be nationally representative of those characteristics. Participants were presented with an informed consent informational sheet prior to receiving the survey and indicated their consent electronically. The survey was soft launched with 200 participants to ensure all questions were answered and that there were no issues with the survey programming. As a quality check, participants who completed the study in less than half the soft launch mean time (12.5 min) were removed from the sample to eliminate participants that were not responding thoughtfully. On average, the survey took 29.4 min to complete. All study procedures were conducted according to the World Medical Association Declaration of Helsinki and to conform to the ICMJE Recommendations for the Conduct, Reporting, Editing, and Publication of Scholarly Work in Medical Journals. All study procedures were approved by the Texas A&M University Institutional Review Board.

#### Materials/measures

Instrument content includes questions to assess time spent in nature, social cognitive measures, attitudes and behavioral capacity, and demographics. When possible, questions were formatted following the same structure and adapted from validated instruments.

### Time spent in nature

Two questions were used to assess time spent in nature. The first question measured the frequency of visits to natural areas. It was adapted from the People and Nature Survey for England-Adults [59]. The question described what types of nature spaces are included and asked, “In the past 12 months, how often, on average have you spent free time outside in green and natural spaces?” Response options included: every day, more than twice a week but not every day, twice a week, once a week, once or twice a month, once every 2–3 months, less often, and never. Some minor changes in the examples were made to increase readability among Americans audiences (e.g., forests for woodlands). The second question, which measures duration rather than frequency, was taken verbatim from the Nature of Americans study [19]. The question asks, “In the typical week, when the weather allows, about how long on average do you spend outdoors in nature?” Response choices included: none, some but less than 30 min, 30 min to an hour, 1–2 h, 2–3 h, 3–4 h, 4–5 h, 5–7 h, and more than 7 h. Both of these aspects of nature exposure are informative to assess, as some research has found frequency and duration to be associated with different outcomes [60].

### Social cognitive measures

The self-efficacy questions all started with the stem: “How confident are you right now that you could spend at least two hours per week in green or natural spaces if….” Response options included: not at all confident, slightly confident, somewhat confident, very confident, and extremely confident. Intention items started with the stem: “In the next three months, do you intend to….” Response options included: strongly disagree, disagree, neither agree nor disagree, agree, and strongly agree.

### Attitudes and behavioral capacity

Additional scales were included to provide convergent validity for self-efficacy and intentions. These included an Attitudes towards Spending Time in Nature scale, which contains three factors: positive attitudes, negative attitudes, and concerns about spending time in nature [61]. A single factor scale measuring behavioral capacity to spend time in nature was also included [62].

### Socio-demographics

Socio-demographic questions assessed included age, gender, race and ethnicity, marital status, education, income, state of residence, zip code, general health, and the ability to walk for more than 10 min.

#### Participants

In the survey portion of the study, 4414 people clicked on the link to the study. Of these, 3847 provided informed consent and 3120 answered any questions. Overall, 2109 participants (67.6% of those that started) completed the survey and passed the quality check. The participants identified as male (49.6%), female (49.7%), and non-binary (0.7%). The median age was 58.1 years (SD = 17.1). The sample was ethnically diverse with 59.8% white respondents, 18.4% Hispanic, 13.3% Black, and 8.0% mixed and other. Median income was $50,000–59,999 and just over half of the sample was married (51.2%). Education was well distributed with 47.4% having less than a college degree, 35.2% having a two- or four-year college degree, and 17.5% having an advanced degree. Respondents came from all 50 states, Puerto Rico, and D.C. Most respondents (74.5%) were in good to excellent health and could walk 10 min (87.5%) without assistance. The sample had a fairly high level of frequency of exposure to nature with 30.5% going into natural spaces every day, 28.8% more than twice per week, and 16.0% less than once per month. Duration of time spent in nature was less, with 47.3% spending less than an hour per week in nature and 22.3% spending one to two hours per week. Only 30.4% met the recommended threshold of spending two or more hours per week in nature. Table 1 presents the sample demographics.

**Table 1:** Sample demographics (n = 2109)

Variable	M (SD) or %
Gender (% Female)	49.7
Age	58.1 (17.1)
Education	
High school or less	21.7
Some or community college	36.8
Bachelor’s degree	24.1
Graduate or professional degree	17.5
Household income	
Less than $30,000	27.7
$30,000–$49,999	21.5
$50,000–$69,999	16.6
$70,000–$99,999	16.9
$100,000+	17.3
Race/ethnicity	
White, non-Hispanic	59.8
Black, non-Hispanic	13.3
Hispanic	18.4
Other	8.0
General health	
Excellent	15.9
Good	58.6
Fair	22.1
Poor	3.4
In the typical week, when the weather allows, about how long on average do you spend outdoors in nature?	
None	5.5
Some but less than 30 min	16.8
30 min to an hour	25.0
1–2 h	22.3
2–3 h	11.0
3–4 h	6.4
4–5 h	5.5
5–6 h	3.0
More than 7 h	4.6
In the last 12 months, how often, on average have you spent your free time outside in green and natural spaces?	
Every day	30.5
More than twice a week, but not everyday	28.8
Twice a week	8.8
Once a week	9.3
Once or twice a month	6.6
Once every 2–3 months	2.5
Less often	4.9
Almost never	8.6

#### Data analyses

In phase five of the study, we conducted item analysis. Survey items with correlations of > .70 with another item were removed to reduce collinearity. Items with extreme distribution characteristics, such as a non-central mean, restriction in range, skewness, and kurtosis, were identified and eliminated.

In phase six, the factor dimensionality of the scales was examined. To facilitate both exploratory and confirmatory analyses, we used the split-half procedure, in which the sample was randomly divided in half. The first half of the sample was selected for exploratory analysis. An exploratory principal components analysis (PCA) was conducted on the matrix of item intercorrelations generated from the first half of the sample using pair-wise deletion. The number of components retained was determined by comparing the results of two procedures (scree procedure and parallel analysis method) that have been shown to be valid predictors of the correct dimensionally of an item set [63]. In some cases, the scree procedure [64] may over extract factors, and for this reason the Parallel Analysis tables developed by Lautenschlager [65] were also used [66]. Orthogonal (varimax) rotations were examined. Items loading less than 0.50 on a factor were removed. Items loading > 0.30 on multiple factors were also removed to reduce collinearity across the subscales.

In phase seven, following the PCA conducted on the first half of the data, confirmatory factor analysis (CFA) was performed on the other half of the data to evaluate the latent structure and provide support for construct validation CFA provides a rigorous test of the proposed scales through testing how well the measures’ variables or items represent the constructs [67]. Indicators were specified and parameters estimated with a maximum likelihood technique using STATA 15.0. Evaluation of a CFA requires an assessment of overall fit to the data [68]. In phase seven CFA was conducted to establish convergent validity through the common variance the items on the construct shared with the latent construct. Hair et al. [68] recommends all factor loadings should be statistically significant with loadings of at least 0.50 or higher to represent convergent validity. Fornell et al. [69] indicates parameter estimates 0.70 or higher are considered acceptable where the amount of information shared with a latent construct is greater than the error variance. Further, the CFA allows for measurement of average variance extracted (AVE). Hair et al. [68] suggests that AVE should be above .50. Reliability is the third criterion of convergent validity, Joerskog Rho indicates construct reliability; values higher than 0.7 indicate internal consistency, which represents all of the items of the scale consistently measuring the same latent construct [68].

In phase eight, reliability of scales was measured using Cronbach’s alpha. In general, an alpha > .70 is considered to be a good measure of internal consistency [70]. If the overall alpha was below .70, individual items were examined to assess if removing the item would increase the overall alpha of the scale. In phase nine to test criterion validity, the relationships between self-efficacy, intentions, and time spent in nature were examined using one-way ANOVAs.

## Results

In phase one, the research team generated 50 unique items for self-efficacy and 24 unique items for intentions. In phase two, the item set for self-efficacy was reduced to 32 items and intentions was reduced to 12 items. Twelve participants pre-tested items during cognitive interviews (phase three) and were diverse in gender, race and ethnicity, geography, age, and educational attainment. The pre-testing phase further reduced the self-efficacy set to 21 items and the intentions set to nine items.

After conducting the national survey, item quality was assessed. All self-efficacy items had good variance and were retained. In bivariate correlations, two items (“you feel stress” and “you feel depressed”) had a correlation of .715 and two additional items (“you are busy” and “you have a lot of work to do”) had a correlation of .707. The items with less variance and higher skewness (“you feel depressed” and “you have a lot of work to do”) and were removed. All other items had correlations less than .70 with the other items. Next, items were assessed relative to the amount of time spent in nature. Five items had small relationships with time spent in nature and were removed resulting in 14 retained items. All intentions items had good variance and were retained. In bivariate correlations, two items (spend more time outside and spent more time in nature) a correlation of .721. Spend more time outside had less variance and higher skewness and was removed leaving 8 items.

In phase six, the PCA was assessed on a randomly selected first half of the sample (*n* = 1607). Eigenvalues for two factors were greater than one (7.69, 1.13) indicating two factors using the Scree procedure [64] and one using Lautenschlager’s tables [65]. Both solutions were investigated. The two-factor solution had several items load on both factors and was uninterpretable. The one factor solution had all items load > 0.60 accounting for 54.9% of the variance. Intentions had only one eigenvalue greater than one (4.82) indicating a one factor solution for both factor extraction methods. All items loaded higher than 0.60 and the factor accounted for 60.3% of the variance.

In phase seven, convergent validity was established by the CFA. Standardized loadings for items measuring self-efficacy ranged from .616 to .779 and were significant (*p* < .001). Further, the CFA demonstrated that the average variance extracted (AVE) from items was .513, above the threshold suggested by Hair et al. (2010), further demonstrating convergent validity. Reliability was the third criterion of convergent validity assessed. The Joreskog Rho construct reliability [68] was .913. A score higher than 0.7 indicates internal consistency, which represents all of the items of the scale consistently measuring the same latent construct [68]. Following the CFA, fit statistics were assessed and model testing indicated an adequate fit to the data (CFI = .906, TLI = .889, SRMR = .051, RMSEA = .097). These data can be found in Table 2. The items measuring intentions ranged from .673 to .814 and were significant (*p* < .001). The AVE was .537, indicating convergent validity, and the Rho was .902 demonstrating construct reliability. The tests for goodness of fit indicated model achieved a mediocre fit to the data (CFI = .914, TLI = .880, SRMR = .053, RMSEA = .135).

**Table 2:** Confirmatory factor analysis results

Constructs and measurement item	Standardized loading^ad^	Error variance^b^	Indicator reliability
First-order loadings			
Self-efficacy (α = .919; ρ = .933; AVE = .584)^c^			
It is really hot outside	.72	.012	.518
It is really cold outside	.69	.012	.476
It is raining or snowing	.68	.012	.462
Daylight hours are shorter	.74	.011	.548
You are busy	.75	.010	.563
You feel stressed	.73	.011	.533
Nature is far away	.70	.012	.490
You feel tired	.78	.010	.608
There are no people around	.65	.014	.423
You have no one to go with	.72	.011	.518
You are in pain	.72	.011	.518
You lack transportation to natural areas	.68	.012	.462
It feels unsafe	.62	.014	.384
There is an expense involved (like a park pass or entrance fee)	.62	.014	.384
Intentions (α = .91; ρ = .90; AVE = .54)^c^			
Spend more time at neighborhood and community parks	.70	.012	.490
Spend at least two hours per week outside	.67	.013	.449
Visit state or national parks	.76	.010	.578
Schedule trips to natural areas	.81	.009	.656
Go on a hike	.73	.012	.533
Go on a walk outdoors	.68	.013	.462
Visit water recreation areas (i.e. lakes, oceans)	.71	.012	.504
Spend more time in nature	.79	.010	.624

^a^Entries are standardized values; all statistically significant (*p* < .01)

^b^Error variance entries are standardized

^c^α = Cronbach’s alpha of reliability; ρ = composite construct reliability; AVE = amount of variance extracted. The average variance estimates (AVEs) ranged between 0.591 and 0.821

^d^Values exceeded the 3.26 cutoff

In phase eight, Cronbach’s alpha for self-efficacy was 0.93 and 0.91 for intentions. The correlation between the two scales was 0.56 (*p* < .001). In phase nine, both scales showed significant differences and eta-squares > .20 indicating large effect sizes across frequency and time spent outside. These results are displayed in Tables 3 and 4. Convergent and divergent validity with demographics, behavioral capacity, and attitudes towards spending time in nature were then assessed. Self-efficacy and intentions were moderately correlated (r = .56, *p* < .001). Self-efficacy was significantly, positively correlated with positive attitudes towards spending time in nature (r = .40, *p* < .001) and behavioral capacity (r = .51, *p* < .001) and negatively correlated with negative attitudes towards spending time in nature (r = − .17), concerns about spending time in nature (− .28, *p* < .001), and age (− .07, *p* < .001). Intentions were significantly, positively correlated with positive attitudes (r = .58, *p* < .001) and behavioral capacity (r = .56, *p* < .001) and negatively correlated with negative attitudes (r = − .24, *p* < .001), concerns about nature (− .19, *p* < .001), and age (− .16, *p* < .001). Males reported significantly higher self-efficacy scores than women. However, there were no gender differences on intentions. For race/ethnicity, there were no differences on self-efficacy. For intentions, Hispanic participants scored significantly higher than both Black and White respondents. Both scales were significantly related to general health, with participants in excellent and good health reporting higher levels than those in fair and poor health. These results are displayed in Table 5.

**Table 3:** One-way ANOVA of self-efficacy and intentions by frequency of visits to natural spaces

Frequency of visits to natural areas and greenspaces	Self-efficacy	Intentions
	F (7,2060) = 57.3, *p* < .001, η^2^ = 0.22	F (7,2090) = 127.3, *p* < .001, η^2^ = 0.30
	M (SD)	M (SD)
Almost never	1.79 (0.81)	2.13 (0.97)
Less than every 2–3 months	2.16 (0.80)	2.61 (0.87)
Once every 2–3 months	2.13 (0.77)	3.09 (0.93)
Once or twice a month	2.28 (0.74)	3.19 (0.76)
Once a week	2.37 (0.78)	3.34 (0.83)
Twice a week	2.66 (0.77)	3.59 (0.75)
More than twice a week, but not everyday	2.83 (0.82)	3.73 (0.80)
Everyday	3.20 (0.90)	3.92 (0.78)

**Table 4:** One-way ANOVA of self-efficacy and intentions by times per week spent in natural spaces

Time per week in natural areas and greenspaces	Self-efficacy	Intentions
	F (8,2057) = 49.8, *p* < .001, η^2^ = 0.22	F (8,2087) = 71.9, *p* < .001, η^2^ = 0.25
	M (SD)	M (SD)
None	1.75 (0.84)	2.14 (1.07)
Some but less than 30 min	2.14 (0.77)	2.86 (0.92)
30 min to an hour	2.60 (0.81)	3.53(0.86)
1–2 h	2.83 (0.85)	3.70 (0.77)
2–3 h	3.08 (0.82)	3.82 (0.77)
3–4 h	3.17 (0.89)	3.91 (0.82)
4–5 h	3.32 (0.89)	3.90 (0.75)
5–7 h	3.21 (0.87)	4.07 (0.65)
More than 7 h	3.45 (0.91)	4.08 (0.82)

**Table 5:** Convergent and divergent validity of self-efficacy and intentions scales

Gender	Self-efficacy	Intentions
	t(2041) = 7.16, *p* < .001, d = .32	t(2071) = 0.76, n.s
	M (SD)	M (SD)
Males	2.86 (0.96)	3.52 (0.96)
Females	2.56 (0.89)	3.48 (0.89)
Race/Ethnicity	F (3,2003) = 1.2, n.s	F (3,2032) = 8.2, *p* < .001, η^2^ = 0.01
White, non-Hispanic	2.68 (0.94)	3.44 (0.90)
Black	2.75 (0.94)	3.46 (0.99)
Hispanic	2.78 (0.90)	3.73 (0.92)^1^
Other	2.68 (0.99)	3.55 (0.90)
General health	F (3,2058) = 88.1, *p* < .001, η^2^ = 0.11	F (3,2088) = 58.8, *p* < .001, η^2^ = 0.08
Excellent	3.28 (0.98)^2^	3.90 (0.86)^2^
Good	2.74 (0.88)	3.55 (0.90)
Fair	2.33 (0.84)	3.16 (1.00)
Poor	1.98 (0.81)	2.73 (1.20)

^1^Hispanic participants scored significantly higher than White and Black participants, Tukey HSD

^2^All groups are significantly different from each other, Tukey HSD

## Discussion

The goal of this study was to develop reliable and valid measures for self-efficacy and intentions to spend time in nature. Having a better understanding of cognitive factors that are associated with individuals spending time in nature will support health promotion and evaluation efforts. The study followed gold standard guidelines proposed by Boateng et al. [58]. For both scales, a one-factor solution was found that was validated in a confirmatory factor analysis. Both scales had excellent levels of internal consistency. The scales were moderately correlated with each other and had a strong relationship with TSN.

Implications for measuring the relationship of nature to health based on these findings include important insights into the ways in which perceived affective responses to time in nature may impact propensity for frequency and duration of exposure to natural spaces. In general, total duration of exposure was positively associated with both self-efficacy and intentions to spend time in nature. Thus, recommendations for developing interventions to increase time spent in nature based on findings from this study include a focus on measuring and increasing confidence to spend time in nature in a variety of situations while also addressing intentions to spend time in a variety of greenspaces. Strategies will therefore include a variety of different approaches, depending upon the specific aspects that are the focus of an intervention. A systematic review and meta-analysis found that successful strategies to increase self-efficacy for physical activity included: action planning, time management, prompt self-monitoring of behavioral outcomes, and planning social support and social change [71]. For example, increasing self-efficacy in nature may be accomplished through observation learning or guided experiences that increase connectedness to nature or sense of place, while intentions might be effectively increased through goal setting, planning, addressing barriers and consciousness raising [72].

There were several interesting findings related to the scales and demographics. While nature exposure is shown to be beneficial to healthy aging, both self-efficacy and intentions were negatively correlated with age [73]. Older adults may have mobility or safety concerns that reduce their intentions and self-efficacy for spending time in nature. In the U.S., less than 10% of park users are older adults and may benefit from parks constructed with older adults in mind [74, 75]. Similar findings were found for general health, with healthier people reporting higher intentions and self-efficacy. For gender, while there was no difference in intentions, males reported higher self-efficacy. A recent study found that although females reported being more connected to nature and preferred outdoor environments for recreation, they were less likely to participate in nature-based recreation [24]. This may be due in part to a difference in self efficacy. Racial and ethnic differences were limited; for self-efficacy no differences were found and intentions were only higher for Hispanic respondents. While more research into this is needed, Taylor [76] likewise found little differences in racial and ethnic groups on connectedness to nature and landscape preferences.

Positive attitudes towards spending time in nature were strongly related to intentions, while negative attitudes and concerns about nature had smaller relationships. While self-efficacy also had a strong relationship with positive attitudes, concerns about being in nature were also correlated. This provides preliminary insight into the value of increasing positive attitudes while providing strategies to address concerns about nature. Negative attitudes seem to play less of a role in intentions or self-efficacy. This is in line with behavioral capacity which was strongly related to both scales and may be a tangible pathway to improve self-efficacy and intentions [72].

Over the past few years, interventions to increase time in nature have become more popular. However, these have been based on adding plants and gardens to indoor and urban areas, physician recommendations for nature contact (e.g. ParkRX), or place-based programming (e.g. community gardens) [43, 60]. To date, there is a lack of theory-based behavioral change interventions focused on individuals, families, or other social groups. This study offers progress toward the provision of measures that will serve as the foundation for interventions about self-efficacy and intentions to spend time in nature. The resulting reduction in items based on factor loadings further created succinct and user-friendly measures that can be applied across a variety of academic and practical contexts.

This study has several limitations. While the sample came from across the United States and was representative of the US population on gender and race, the average age of the respondents tended to be older than the population median. Since the survey was collected via the internet, no validation of time spent in nature was possible but the study did use validated measures of nature exposure from the United States and the United Kingdom. With an internet-based study, people without internet access or the ability to read and write in English were excluded. The self-efficacy scale does differ a bit from Bandura’s [77] recommendations which included using a 10-point Likert scale and using responses worded as certainty rather than confidence. However, in the health promotion literature, most self-efficacy scales have used a 5-point Likert scale with confidence as the prompts [78, 79].

In conclusion, this study resulted in reliable and valid measures of self-efficacy and intentions to spend time in nature. The measures will be helpful in developing and evaluating theory-based interventions to increase time in nature.

## Data Availability

The data and scales are available by contacting the corresponding author.
